# A Silver Modified Nanosheet Self-Assembled Hollow Microsphere with Enhanced Conductivity and Permeability

**DOI:** 10.3390/molecules29184384

**Published:** 2024-09-15

**Authors:** Fangmin Wang, Xue Dong, Yuzhen Zhao, Zemin He, Wenqi Song, Chunsheng Li, Jiayin Li, Jianfeng Huang, Zongcheng Miao

**Affiliations:** 1 Xi’an Key Laboratory of Advanced Photo-Electronics Materials and Energy Conversion Device, Technological Institute of Materials & Energy Science (TIMES), Xijing University, Xi’an 710123, China; wangfangmin@xijing.edu.cn (F.W.); xdong1208@outlook.com (X.D.); zyz19870226@163.com (Y.Z.); zeminhe315@126.com (Z.H.); songwenqi@xijing.edu.cn (W.S.); 2 Key Laboratory of Advanced Electrode Materials for Novel Solar Cells for Petroleum and Chemical Industry of China, School of Chemistry and Life Sciences, Suzhou University of Science and Technology, Suzhou 215009, China; lichsheng@163.com; 3 School of Material Science and Engineering, Shaanxi University of Science and Technology, Xi’an 710021, China; lijiayin@sust.edu.cn; 4 School of Artificial Intelligence, Optics and Electronics (iOPEN), Northwestern Polytechnical University, Xi’an 710072, China

**Keywords:** electromagnetic shielding, conductive filler, nanosheet structure

## Abstract

The utilization of sheet structure composites as a viable conductive filler has been implemented in polymer-based electromagnetic shielding materials. However, the development of an innovative sheet structure to enhance electromagnetic shielding performance remains a significant challenge. Herein, we propose a novel design incorporating silver-modified nanosheet self-assembled hollow spheres to optimize their performance. The unique microporous structure of the hollow composite, combined with the self-assembled surface nanosheets, facilitates multiple reflections of electromagnetic waves, thereby enhancing the dissipation of electromagnetic energy. The contribution of absorbing and reflecting electromagnetic waves in hollow nanostructures could be attributed to both the inner and outer surfaces. When multiple reflection attenuation is implemented, the self-assembled stack structure of nanosheets outside the composite material significantly enhances the occurrence of multiple reflections, thereby effectively improving its shielding performance. The structure also facilitates multiple reflections of incoming electromagnetic waves at the internal and external interfaces of the material, thereby enhancing the shielding efficiency. Simultaneously, the incorporation of silver particles can enhance conductivity and further augment the shielding properties. Finally, the optimized Ag/NiSi-Ni nanocomposites can demonstrate superior initial permeability (2.1 × 10^−6^ H m^−1^), saturation magnetization (13.2 emu g^−1^), and conductivity (1.2 × 10^−3^ Ω•m). This work could offer insights for structural design of conductive fillers with improved electromagnetic shielding performance.

## 1. Introduction

With the rapid development of fifth-generation (5G) mobile networks, various electronic instruments have become extensively utilized in diverse domains and households [1,2,3]. The electromagnetic compatibility of precision instruments, potential harm to human health caused by electromagnetic radiation, threats to national information security due to electromagnetic information leakage, and a series of associated issues have emerged as significant concerns [4,5,6]. Consequently, the problem of shielding against electromagnetic radiation has garnered considerable attention. Among all strategies aimed at reducing electromagnetic contamination, the utilization of enhanced full-band electromagnetic shielding materials in electrical and electronic instruments has proven to be highly effective [7,8,9].

The total shielding effectiveness (SE) of the material could be obtained by summing the contributions of reflection (R), absorption (A), and multiple reflection (B) in dB, as shown below:

(1) $${\mathrm{SE}}_{\mathrm{dB}}{=R}_{\mathrm{dB}\;}+{\;A}_{\mathrm{dB}}+B_{\mathrm{dB}}$$

(2) $$R_{\mathrm{dB}}=168-10\log_{10}\left(\sigma \mu f^{2}\right)$$

(3) $$A_{\mathrm{dB}}=1.314\;\times \;t(f\sigma \mu {)}^{1/2}$$

(4) $$B_{\mathrm{dB}}=20\log_{10}\left(1\;-e^{-2t(\pi f\mu \sigma {)}^{1/2}}e^{-j-2t(\pi f\mu \sigma {)}^{1/2}}\right)$$

where μ is the relative magnetic permeability, σ is the electrical conductivity, t is the material thickness, and ƒ is the frequency [10,11]. It is obvious that high electrical and magnetic conductivity are essential properties of electromagnetic shielding materials.

As a novel research focus, polymer-based electromagnetic shielding materials typically consist of a polymer matrix and conductive or magnetic fillers, offering the advantages of cost-effectiveness, lightweightness, and ease of processing [12,13,14]. Various types of conductive fillers exist, and their structure, properties, and content directly influence the formation of a conductive network within the matrix, thereby impacting the electromagnetic shielding performance of composite materials. According to the structure of conductive packing, it can be categorized into micro-particle type packing [15,16], high-aspect-ratio structure packing [17,18], lamellar structure packing [19,20] and so on. It has been observed that the incorporation of sheet structures can enhance the performance of polymer-based electromagnetic shielding materials; however, there is still considerable room for improvement in this regard. Recent scientific reports have highlighted the significance of constructing nanosheet self-assembled hollow structures with a large specific surface area as a crucial approach for material modification [21]. Nevertheless, limited research has been conducted on this topic within this field.

Herein, a nanosheet self-assembled hollow sphere structure was constructed. The hollow NiSi spheres with nanosheet self-assembled structures on the surface were reduced at high temperature through the hydrothermal reaction between SiO_2_ and NiCl_2_. To further enhance the packing’s EMI (electromagnetic interference) shielding properties, Ag nanoparticles were used to form an enhanced coating, using electroless plating, because of their high electrical conductivity (Ag/NiSi-Ni) [22]. Importantly, it was found that the magnetic and electrical conductivity could be finely controlled by adjusting the structure of the nanocomposites. The utilization of the silver-loaded nanosheet self-assembled hollow sphere structure enables the attainment of exceptional magnetic and electrical conductivity.

## 2. Results and Discussion

Firstly, SiO_2_ nanospheres were prepared using the Stöber hydrolysis method, while the synthesis principle has been investigated by previous researchers [23,24]. The successful preparation of amorphous SiO_2_ with an average particle size of 375 nm by the Stöber hydrolysis method is demonstrated in Figure S1, exhibiting a narrow particle size distribution and excellent dispersion. The XRD pattern in the lower-left corner of the image indicates that the original SiO_2_ exhibited a broad diffraction peak centered at approximately 22°, which could be attributed to the cristobalite silica phase.

The formation mechanism of NiSi-Ni(OH)_2_ in the hydrothermal process is elucidated as follows: initially, the silica sphere surface undergoes attack by hydroxyl groups, leading to the generation of silicate ions during the reaction [25]. The silicate ion generated in the high-temperature alkaline solution undergoes a reaction with nickel ions, resulting in the formation of nickel silicate that selectively grows on the surface of silica spheres and forms a NiSi shell in situ. As the reaction progresses, the silica core is gradually consumed and hollow NiSiO_x_ spheres are formed. In this process, ammonium chloride plays a crucial role by adjusting the pH value and suppressing the formation of Ni(OH)_2_. Upon complete reaction of SiO_2_ into NiSiO_x_, the remaining Ni^2+^ ions undergo subsequent reactions to form a coating of Ni(OH)_2_ on the surface of the NiSi shell, resulting in the formation of a composite structure known as NiSi-Ni(OH)_2_. According to the energy spectrum analysis conducted by EDS, Figure S2 reveals that the nanoparticles primarily consisted of elements such as Ni, Si, and O.

**Figure 1:**
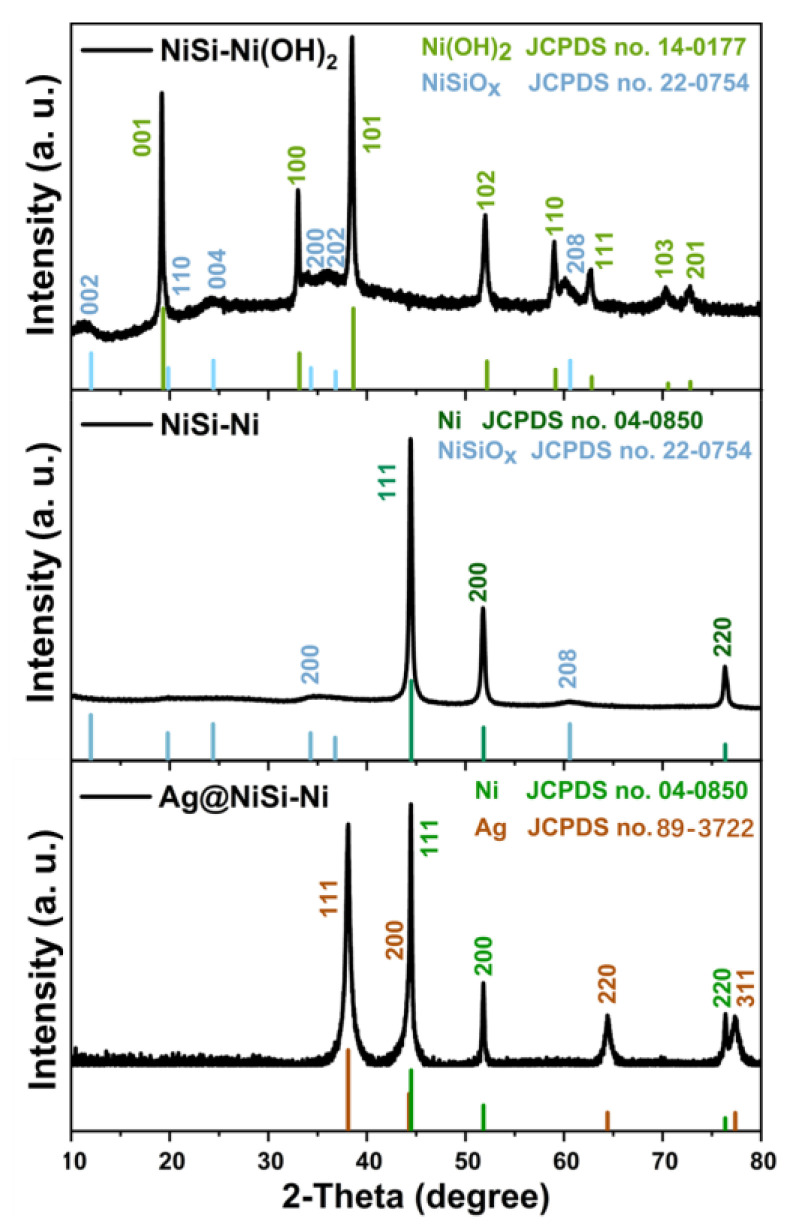
XRD patterns of NiSi-Ni (OH)_2_, NiSi-Ni, and Ag/NiSi-Ni.

The X-ray Diffraction (XRD) patterns of NiSi-Ni(OH)_2_, NiSi-Ni, and Ag/NiSi-Ni are presented in Figure 1. For the XRD pattern of NiSi-Ni(OH)_2_, the peaks of 19.6°, 33.4°, 38.8°, 52.2°, 59.2°, 63.1°, 70.8°, and 72.7° were attributed to the (001), (100), (101), (102), (110), (111), (103), and (201) crystal planes of Ni(OH)_2_ (JCPDS No. 14-0177), respectively. The peaks of 11.9°, 19.7°, 24.3°, 34.2°, 36.7°, and 60.5° corresponded to the (002), (110), (004), (200), (202), and (208) crystal faces of Ni_3_Si_2_O_5_(OH)_4_ (JCPDS No. 22-0754), respectively. The diffraction peaks observed in the analysis indicated that they were a result of the combination of Ni(OH)_2_ and NiSiO_x_, providing evidence for the successful synthesis of the NiSi-Ni(OH)_2_ composite. The XRD patterns of NiSi-Ni at 44.3°, 51.9°, and 76.4° corresponded to the (111), (200), and (220) crystallographic planes of nickel (JCPDS No. 04-0850). The peaks of 34.3° and 60.7° corresponded to the (200) and (208) crystal planes of Ni_3_Si_2_O_5_(OH)_4_; however, these peaks were not prominently visible in the spectrum. It is evident that Ni(OH)_2_ underwent reduction to elemental nickel during the hydrogen reduction process, while retaining the existence of a NiSi shell structure. Nevertheless, due to the high intensity of the diffraction peak from pure nickel, there is limited evidence for strong diffraction phenomena associated with NiSiO_x_. The XRD pattern of Ag/NiSi-Ni exhibited three prominent Ni peaks, along with additional diffraction peaks at 38.0°, 44.3°, 64.5°, and 77.4°, corresponding to the (111), (200), (220), and (311) crystallographic planes of Ag (JCPDS No.89-3722), respectively. The results demonstrated the successful introduction of Ag into the system through electroless silver plating. Figure S3 presents the EDS energy spectrum analysis of the Ag/NiSi-Ni composite, revealing the presence of Ni, Si, O, and Ag elements, thereby confirming the incorporation of Ag. In order to analyze the silver load in the Ag/NiSi-Ni, X-ray fluorescence (XRF) tests were performed on the sample (Figure S6). The test results showed that the silver content of Ag/NiSi-Ni was 40.7%.

**Figure 2:**
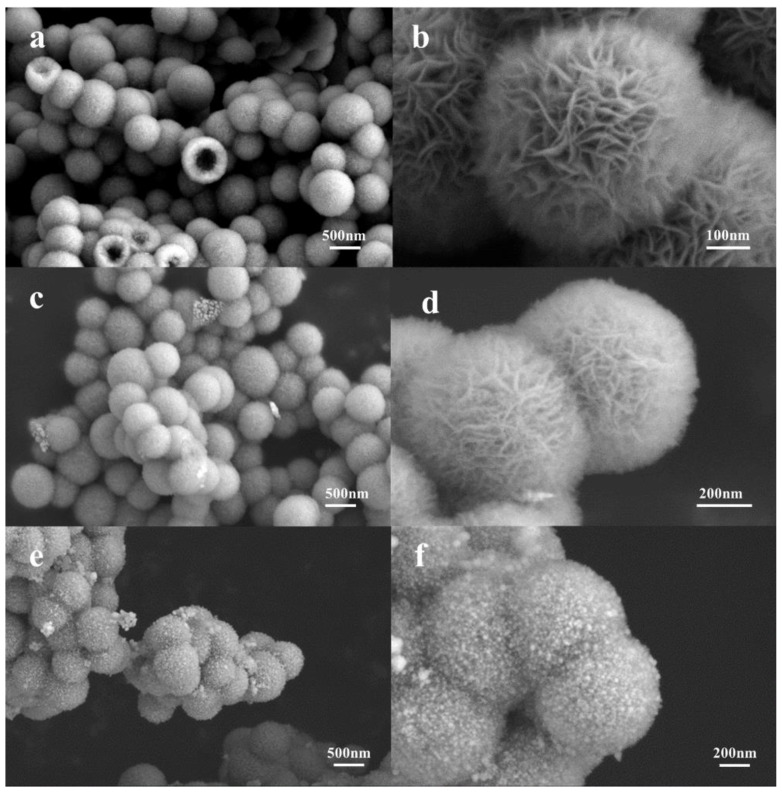
The SEM images of NiSi–Ni(OH)_2_ (**a**,**b**), NiSi–Ni (**c**,**d**), and Ag/NiSi-Ni (**e**,**f**).

The scanning electron microscopy (SEM) images in Figure 2a reveal that the average particle size of NiSi-Ni(OH)_2_ spheres was 500 nm, with a visible hollow structure observed in some broken spheres. Most of the spheres had intact and unbroken shells. The diameter of the hollow region measured approximately 325 nm, which closely corresponds to the initial diameter of SiO_2_ involved in the reaction. This finding provides further evidence supporting the formation mechanism of the NiSi hollow shell. The high-resolution scanning electron microscopy (Figure 2b) revealed the presence of a flower-shaped Ni(OH)_2_ structure, formed through the self-assembly of nanosheets. This structure exhibited uniform attachment onto the surface of the hollow shell, resulting in a well-defined hierarchical architecture. The thickness of the Ni(OH)_2_ nanosheet was about 5 nm, and the thickness of the Ni(OH)_2_ layer was about 100 nm. Figure 2c,d are SEM images of NiSi-Ni, which were obtained from the reduction of NiSi-Ni(OH)_2_ by H_2_. Upon comparison, it is evident that there was no significant alteration in the microscopic morphology of the samples following H_2_ reduction. In other words, the flower-like structure of Ni(OH)_2_ remained unchanged during its conversion process. Figure 2e shows images of Ag/NiSi-Ni, which was obtained after the silver plating of NiSi-Ni. After electroless silver plating, nanosized Ag particles were coated on the surface of the particles. Figure 2f reveals the uniform embedding of Ag nanoparticles with a fine particle size into the flower-like lamellae structure.

To investigate the variations in particle size of samples at different stages during the preparation process, an analysis of particle size distribution statistics was conducted. Figure S4 shows the particle size distribution of SiO_2_, NiSi-Ni (OH)_2_, NiSi-Ni, and Ag/NiSi-Ni nanoparticles. In the overall trend, there was a gradual increase in particle size, particularly observed during the hydrothermal synthesis of NiSi-Ni(OH)_2_ from SiO_2_ and the electroless Ni plating of Ag/NiSi-Ni from NiSi-Ni, where the average particle size increased from 375 nm to 500 nm and from 540 nm to 650 nm, respectively.

**Figure 3:**
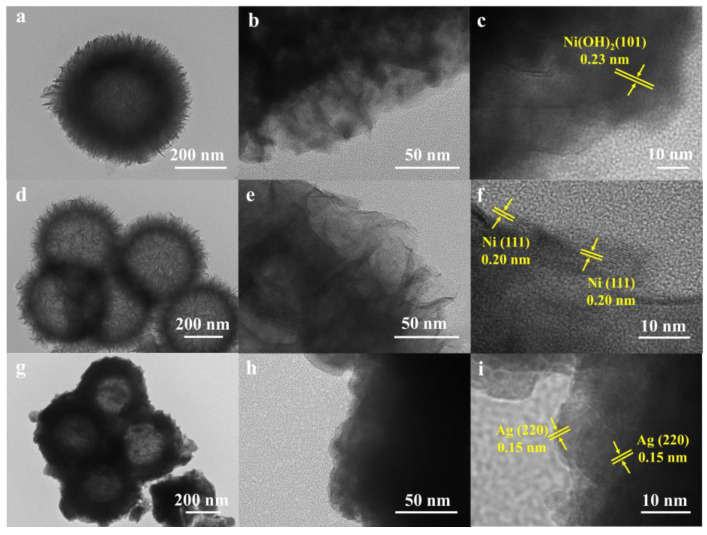
The TEM and HRTEM images of NiSi–Ni(OH)_2_ (**a**–**c**); NiSi–Ni (**d**–**f**); and Ag/NiSi-Ni (**g**–**i**).

Transmission electron microscopy (TEM) testing was employed to elucidate the structural characteristics of the sample more effectively. The TEM and high resolution TEM (HRTEM) images of NiSi-Ni(OH)_2_ are presented in Figure 3a–c, revealing a distinct nanosheet self-assembly hollow sphere structure. Notably, the HRTEM image (Figure 3c) clearly displays lattice fringes with a spacing of 0.23 nm, corresponding to the (101) planes of Ni(OH)_2_. Additionally, Figure 3d–f and Figure 3g–i depict the TEM and HRTEM images of NiSi-Ni and Ag/NiSi-Ni, respectively, demonstrating that both samples maintained their well-preserved self-assembled hollow sphere structures. The flower-like spheres with laminated nanosheets possessed a significantly high specific surface area, ensuring multiple reflections for effective electromagnetic shielding. Simultaneously, the hollow architecture facilitated the occurrence of multiple reflections at material interfaces, thereby enhancing the shielding efficiency. The HRTEM images of NiSi-Ni (Figure 3f) exhibit lattice fringes measuring 0.20 nm, corresponding to the (111) plane of nickel. Figure 3g–h depict the TEM image subsequent to silver loading. Upon careful examination of the HRTEM image, distinct lattice fringes attributed to the (220) planes of silver are clearly discernible. The EDS elemental mappings (Figure S5) of the Ag/NiSi-Ni confirmed the homogeneous distribution of Ag, Ni, and Si elements.

**Figure 4:**
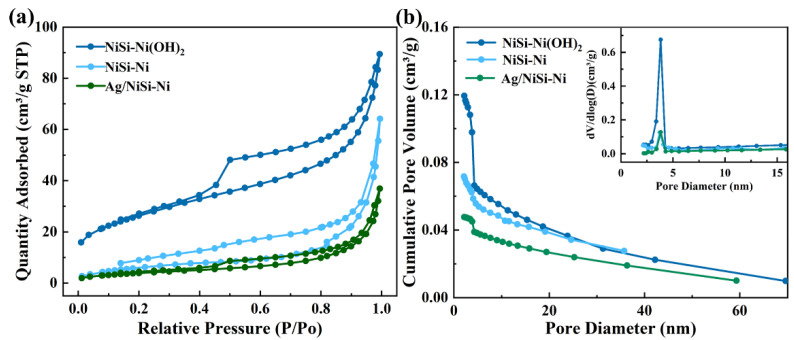
Nitrogen adsorption, desorption isotherms (**a**), and pore diameter distributions (**b**) of NiSi-Ni (OH)_2_, NiSi-Ni, and Ag/NiSi-Ni.

The nitrogen adsorption–desorption isotherms of the product were measured to investigate the specific surface area and pore size distribution, as depicted in Figure 4. All isotherms exhibited a characteristic IV-type classification, which is indicative of mesoporous materials. The Brunauer–Emmett–Teller (BET) specific surface areas of NiSi-Ni(OH)_2_, NiSi-Ni, and Ag/NiSi–Ni were 94.2, 23.5, and 14.6 m^2^g^−1^, respectively. It suggests that the self-assembly of thin nanosheets led to a significantly enhanced specific surface area. By using the Barrett–Joyner–Halenda (BJH) method, the pore size distributions were calculated via the desorption branch of the isotherms. In Figure 4b, the pore sizes of NiSi-Ni(OH)_2_, NiSi-Ni, and Ag/NiSi–Ni were measured to be 6.0 nm, 8.3 nm, and 10.2 nm, respectively. The BJH desorption cumulative pore volumes were determined to be 0.019, 0.076, and 0.048 cm^3^g^−1^ for the respective samples. The Ag/NiSi-Ni composite exhibits promising potential as an optimal candidate for electromagnetic shielding material, considering its specific surface area, pore size distribution, and pore volume [26,27].

**Figure 5:**
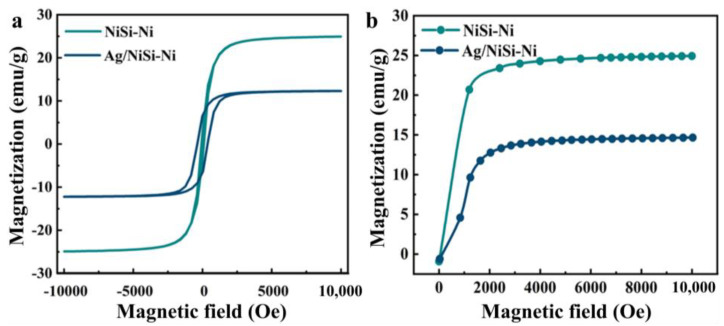
Hysteresis loops (**a**) and initial magnetization curves (**b**) of NiSi-Ni and Ag/NiSi-Ni.

The hysteresis loops and initial magnetization curves of the prepared NiSi-Ni and Ag/NiSi-Ni composites were subsequently examined. As depicted in Figure 5a, both composites exhibited a characteristic S-shaped curve in their respective hysteresis loops. In the first quadrant, the magnetization exhibited a rapid increase, followed by a saturation effect, as both positive and external magnetic fields intensified. The hysteresis loop in the third quadrant initially experienced a steep decline, which then leveled off with an increasing strength of the reverse external magnetic field. The external magnetic field induced the formation of spin magnetic moment through electron spins located outside the nucleus of nickel atoms, while the orbital motion around the nickel nucleus generated an orbital magnetic moment. The resultant combined magnetic moment aligned with the direction of the external magnetic field. The material demonstrated ferromagnetic behavior through its magnetization in the presence of an external magnetic field, thus exhibiting the characteristic hysteresis loops observed in ferromagnetic materials [28,29]. Figure 5a shows that the saturation magnetization of NiSi-Ni and Ag/NiSi-Ni were 24.8 emu g^−1^ and 13.2 emu g^−1^, respectively. The coercivity was 3.0 Oe and 383.4 Oe, respectively. This work demonstrated clear advantages in terms of coercivity when compared to other materials (Table S1). The saturation magnetization decreased and the coercivity increased as a result of electroless silver plating. Due to the diamagnetic properties of silver, it underwent reverse magnetization when subjected to an external magnetic field, thereby diminishing the saturation magnetization of the composite material. The introduction of Ag nanoparticles simultaneously enhanced the formation of grain boundaries, thereby augmenting the blocking effect on magnetic domain movement and resulting in an increased coercivity [30]. Figure 5b shows that the initial permeability of NiSi-Ni and Ag/NiSi-Ni were 3.7 × 10^−6^ H m^−1^ and 2.1 × 10^−6^ H m^−1^, respectively.

**Figure 6:**
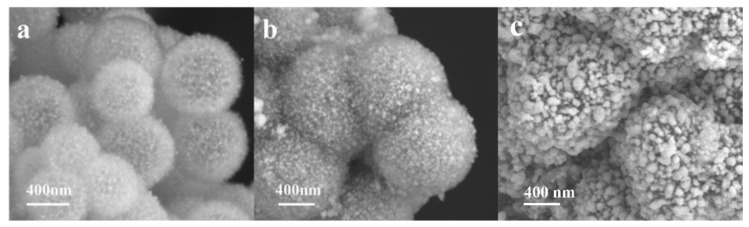
The SEM images of Ag/NiSi–Ni-1 (**a**), Ag/NiSi–Ni-2 (**b**), and Ag/NiSi–Ni-3 (**c**).

**Figure 7:**
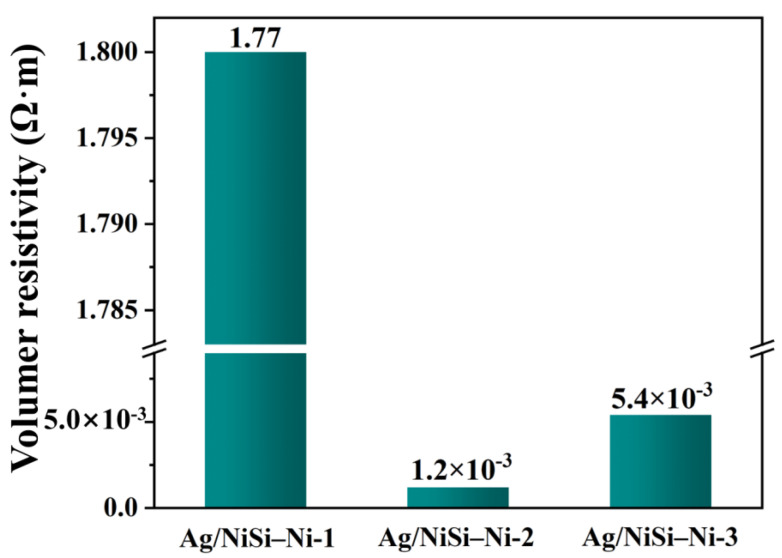
Volume resistivity of Ag/NiSi–Ni–1, Ag/NiSi–Ni–2, and Ag/NiSi–Ni–3.

In order to characterize the effect of the amount of silver plating on the conductivity of Ag/NiSi-Ni composites, diverse samples were prepared by changing the concentration of silver ammonia solution. The composite samples of NiSi-Ni electroless silver plating were named Ag/NiSi–Ni-1, Ag/NiSi–Ni-2, and Ag/NiSi–Ni-3, corresponding to the silver ammonia solution concentrations of 0.15 g L^−1^, 0.35 g L^−1^, and 0.7 g L^−1^, respectively. As shown in Figure 6, there were significant differences in the silver morphology that existed in the system, due to the difference in silver content. With the increase in silver content, silver nanoparticles exhibited dispersion within cluster flower-like structures (Ag/NiSi-Ni-1), uniform coating (Ag/NiSi-Ni-2), and overgrown (Ag/NiSi-Ni-3). Considering the resistivity of the three materials depicted in Figure 7, it is evident that the Ag content in Ag/NiSi-Ni-1 was relatively low, resulting in dispersed particles and a comparatively higher resistivity. The Ag nanoparticles were uniformly coated onto the surface of the Ag/NiSi-Ni-2 material, forming a conductive pathway and resulting in a significantly reduced resistivity of 1.2 × 10^−3^ Ω•m [31]. The resistivity of this work exhibited distinct advantages when compared to other materials (Table S2). For Ag/NiSi-Ni-3, the volume resistivity increased slightly instead of decreasing when the Ag content was further increased. From Figure 6c, it was not difficult to find that the reason was that the Ag layer completely wrapped the material, and the inner hollow self-assembly of nanosheets structure was not obvious. The volume resistivity of Ag/NiSi-Ni-3 exhibited a slight increase, instead of the expected decrease, with a further increase in Ag content. The excessive presence of silver powder completely enveloped the inherent sheet self-assembly structure, leading to a reduction in the material’s specific surface area, impeding electron mobility and elevating resistivity [32,33]. An excess of silver content gave rise to an irregular blocky structure with uneven distribution (Figure 6c), resulting in localized current density surpassing acceptable limits and thereby diminishing overall conductivity. Simultaneously, the flake self-assembly structure exhibited a substantial specific surface area that could accommodate more silver particles when the silver content was appropriately balanced, consequently enhancing electrical conductivity [34,35].

**Figure 8:**
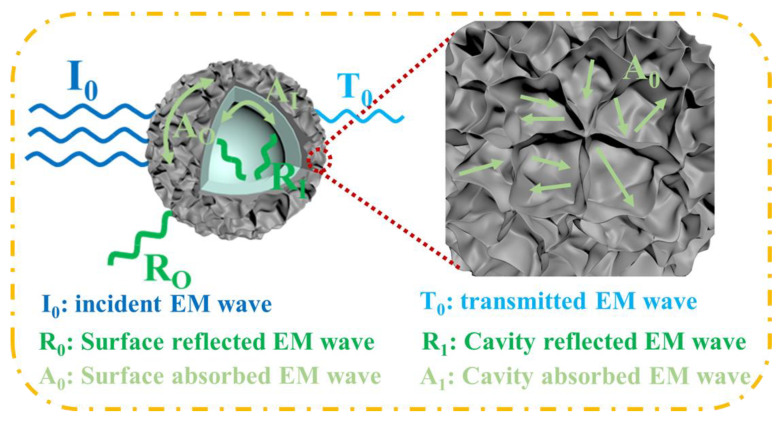
Absorption, reflection, and transmission mechanisms of high-frequency electromagnetic waves in nanosheet self-assembled hollow sphere structure particles.

Combined with the above results, the essential advantages of the Ag/NiSi-Ni structure in this study were revealed. The mechanism of this structure with higher shielding efficiency can be explained by the schematic diagram shown in Figure 8. In the case of hollow nanostructures, both absorption and reflection contributions originated from their outer and inner surfaces, as follows:

(5) $${\mathrm{SE}}_{\mathrm{dB}}{=R}_{\mathrm{dB}}+A_{\mathrm{dB}}+B_{\mathrm{dB}}$$

where R = R_i_ + R_o_ and A = A_i_ + A_o_, R_i_ and A_i_ are the contributions of the reflection and absorption of the inner surface, and R_o_ and A_o_ are the ontributions of the reflection and absorption of the outer surface. When implementing multiple reflection attenuation, the lamellar self-assembled stack structure within the composite material could significantly amplify the occurrence of multiple reflections, thereby effectively enhancing its shielding performance. The hollow structure simultaneously facilitated multiple reflections of incident electromagnetic waves at the material interface, thereby enhancing the shielding efficiency. Theoretically, this structural design exhibited distinct advantages over conventional materials for electromagnetic wave shielding [36].

## 3. Materials and Methods

### 3.1. Material Preparation

Tetraethyl orthosilicate (TEOS), nickel (II) chloride hexahydrate (NiCl_2_·6H_2_O), NH_4_Cl, NH_3_·H_2_O, silver nitrate, formaldehyde (HCHO), and ethanol were purchased from Aladdin Chemical Reagent Co., Ltd. (Shanghai, China) at an analytical grade and used as received.

#### 3.1.1. Synthesis of NiSi-Ni

In a typical process, SiO_2_ spheres with an average diameter of approximately 300 nm were synthesized using the Stöber hydrolysis method.

The SiO_2_ (0.1 g) was prepared via Stöber hydrolysis, a commonly employed method for its synthesis. To obtain solution A, the dispersed SiO_2_ was mixed with 20 mL of deionized water and subjected to ultrasound treatment for 1 h. Solution B was formed by vigorously stirring 1.2 g NiCl_2_·6H_2_O, 10 mmol NH_4_Cl, and 2 mL NH_3_·H_2_O in 20 mL deionized water for a duration of 10 min. The mixture of Solution A and B was prepared by adding solution B dropwise to solution A. The resulting mixture was vigorously stirred for 30 min and subsequently transferred into a hydrothermal reactor. Following a hydrothermal reaction at 120 °C for 24 h, the resulting green precipitate was thoroughly washed with deionized water and ethanol until reaching a neutral pH. The product was subsequently subjected to vacuum drying at a temperature of 70 °C for a duration of 12 h. The resulting synthesized product was designated as NiSi-Ni(OH)_2_. Finally, the thermal reduction of the prepared precursors under an N_2_/H_2_ (N_2_:H_2_ = 95:5 vol %) atmosphere in a tubular oven at 500 °C for 2 h led to the formation of NiSi-Ni composites.

#### 3.1.2. Synthesis of Ag/NiSi-Ni

**Figure 9:**
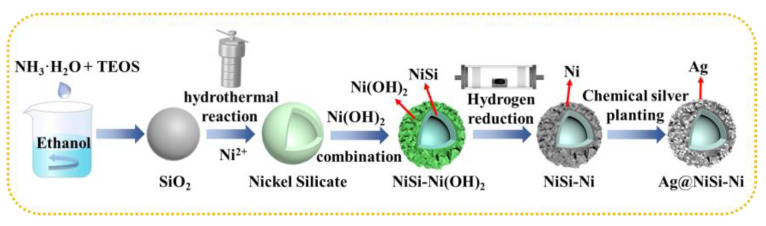
Schematic illustration of the preparation process of Ag/NiSi-Ni.

The silver ammonia solution was prepared by adding 80 mL of deionized water to a beaker containing a specific quantity of silver nitrate (15 mg, 35 mg, 70 mg), followed by the gradual addition of 20 mL of 25 wt% ammonia, while adjusting the pH to 13 using sodium hydroxide. Subsequently, a sequential addition of 95 mL anhydrous ethanol, 4 mL deionized water, and 2.5 mL formaldehyde solution at a concentration of 37 wt% was performed into the beaker containing NiSi-Ni particles, followed by ultrasonic dispersion for a duration of 15 min. Subsequently, the specimen was immersed in a water bath maintained at 25 °C, followed by the addition of the prepared silver ammonia solution within a time frame of 20 min. The mixture was continuously stirred for a duration of 2 h. Upon completion of the reaction, precipitation separation was conducted through thorough cleansing with deionized water and ethanol to achieve neutralization. Finally, Ag/NiSi-Ni was obtained after drying. The schematic representation of this simplified sample preparation process is illustrated in Figure 9.

### 3.2. Methods

Morphological data and energy dispersive spectra (EDS) were obtained using a field emission scanning electron microscopy (FE-SEM) (GeminiSEM500, Shanghai, China) and a transmission electron microscopy (TEM) (JEOL JEM2100, Tokyo, Japan). X-ray Diffraction (XRD) measurements were carried out using a PANalytical X’pert MPDPro (Almelo, The Netherlands) diffractometer with a Cu Ka radiation source (40 kV, 40 mA). Brunauer–Emmett–Teller (BET) surface areas and pore size distributions were obtained at −196 °C (liquid nitrogen temperature) using an ASAP 2020 (Norcross, GA, USA) instrument. Electric conductivity properties were measured using a HP RLC4284A Bridge. Magnetic properties were measured using a TF-CIB static hysteresis loop measurement instrument. The X-ray fluorescence (XRF) technique employed a BRUKER (S8 TIGER) wavelength dispersive X-ray fluorescence spectrometer equipped with an RHX-ray tube, a 4 kW generator, and an 8-bit crystal converter. The Si, Ni, Ag, and Rh tubes operated at 50 kV and 50 mA.

The resistivity of the composite powder was determined using powder bulk resistivity measurement. In this test, a specific quantity of conductive powder to be measured was added to the sample reservoir, followed by the installation of the load device. Once the circuit was switched on, the resistance indicating value was recorded as soon as it stabilized, representing the stacking resistance R (Ω) of the sample. The bottom area of the sample reservoir was denoted by S (m^2^), and L (m) represented the height at which the powder was stacked. Equation (6) illustrates the calculation method for determining ρ, which represents the resistivity of powdered packing, as follows:

(6) $$\rho =R(\frac{S}{L})$$

## 4. Conclusions

In this paper, a novel electromagnetic shielding filler with a nanosheet self-assembled hollow sphere structure was controlled. It exhibited excellent electrical and magnetic conductivity. The initial permeability, saturation magnetization, and conductivity of optimized Ag/NiSi–Ni nanocomposites were obtained as 2.1 × 10^−6^ H m^−1^, 13.2 emu/g, and 1.2 × 10^−3^ Ω•m, respectively. When implementing multiple reflection attenuation, the incorporation of a hollow structure and a self-assembled stack structure could significantly amplify the occurrence of multiple reflections, thereby effectively enhancing the shielding performance. Simultaneously, the incorporation of silver particles enhanced conductivity and further augments shielding efficiency. This study provides a new idea for the preparation of electromagnetic shielding filler. Hollow spherical composite powder is employed as a conductive filler, which also possesses distinctive advantages, such as its lightweight nature within the aerospace domain.

## Data Availability

Data are available upon request from the corresponding authors.

## Supplementary Materials

The following supporting information can be downloaded at: https://www.mdpi.com/article/10.3390/molecules29184384/s1. Figure S1. XRD and SEM of SiO_2_ were prepared by the Stöber hydrolysis method.; Figure S2. EDX spectra of NiSi-Ni(OH)_2_. Figure S3. EDX spectra of Ag/NiSi-Ni.; Figure S4. Particle size distribution of different nanoparticles.; Figure S5. (a) TEM image of Ag/NiSi-Ni and their EDS elemental mappings of Si(b), Ni(c) and Ag(d) elements; Figure S6. XRF test results of Ag/NiSi-Ni.; Table S1. Comparison of the performance of Coercivity (Oe); Table S2. Comparison of the performance of Resistivity (Ω•m); References [37,38,39,40,41,42,43,44] are cited in Supplementary Materials.
