# Supplementary material for: A Silver Modified Nanosheet Self-Assembled Hollow Microsphere with Enhanced Conductivity and Permeability
Source: Molecules. 2024 Sep 15;29(18):4384. doi: 10.3390/molecules29184384 (PMC11434284; doi:10.3390/molecules29184384)
Supplement: Supplementary file 1 [file molecules-29-04384-s001.zip › molecules-3157207-supplementary.pdf]

Supporting information

A silver-modified nanosheet self-assembled hollow microsphere with enhanced conductivity and permeability

Fangmin Wang [a], Xue Dong [a],Yuzhen Zhao [a], , Zemin He [a], Wenqi Song [a],

Chunsheng Li [b] Jiayin Li [c], Jianfeng Huang [c,*] and Zongcheng Miao [a,d,*]

[a] *Xi'an Key Laboratory of Advanced Photo-Electronics Materials and Energy Conversion Device, Technological Institute of Materials &Energy Science (TIMES), Xijing University, Xi'an, 710123, P, R, China.*

[b] *Key Laboratory of Advanced Electrode Materials for Novel Solar Cells for Petroleum and Chemical Industry of China, School of Chemistry and Life Sciences, Suzhou University of Science and Technology, Suzhou 215009, P, R, China.*

[c] *School of Material Science and Engineering, Shaanxi University of Science and Technology, Xi' an, 710021, P. R. China.*

[d] *School of Artificial Intelligence, Optics and Electronics (iOPEN), Northwestern Polytechnical University, Xi'an, 710072, P.R. China.*

[*] Corresponding authors: huangjf@sust.edu.cn (J. Huang);

miaozongcheng@nwpu.edu.cn (Z. Miao)

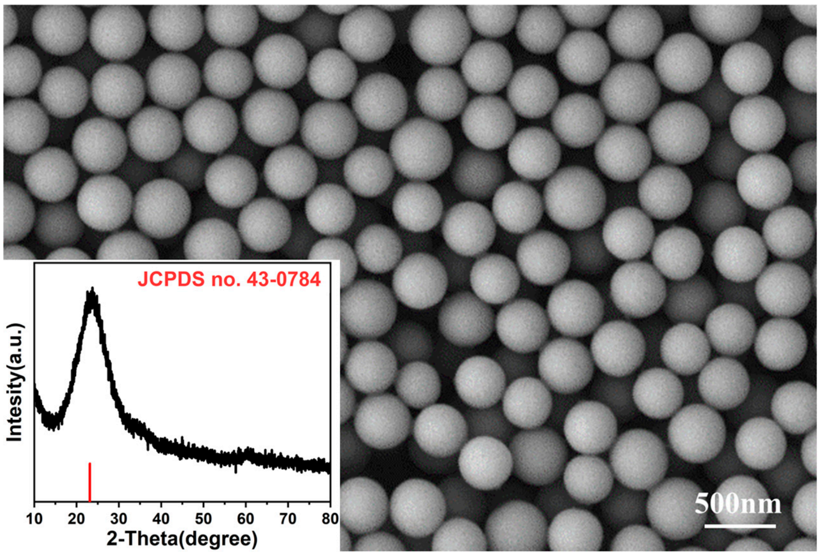

Fig.S1 XRD and SEM of SiO_2 were prepared by the Stöber hydrolysis method.

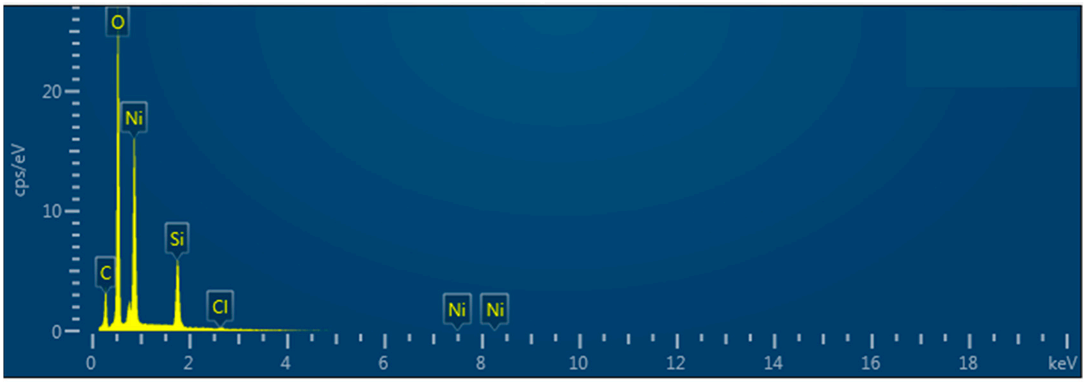

Fig.S2 EDX spectra of NiSi-$Ni(OH)_2$.

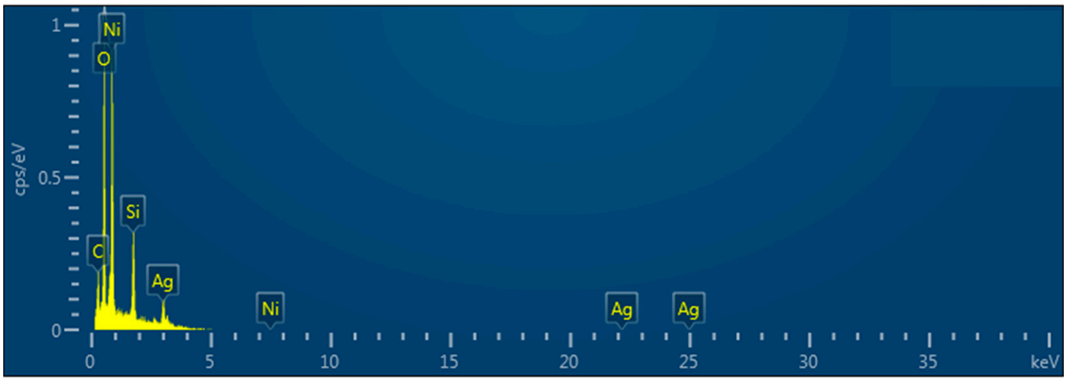

Fig.S3 EDX spectra of Ag/NiSi-Ni.

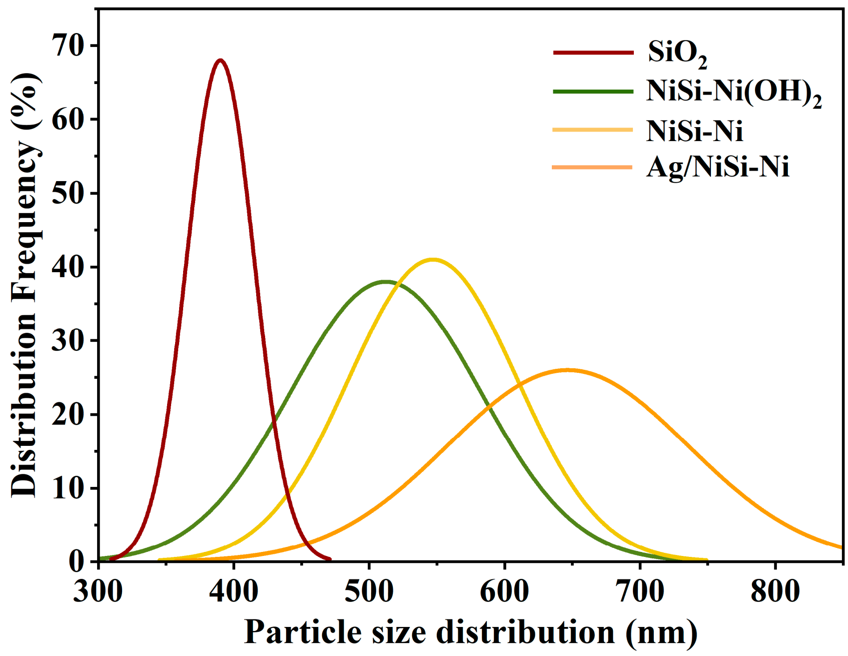

Fig.S4 Particle size distribution of different nanoparticles.

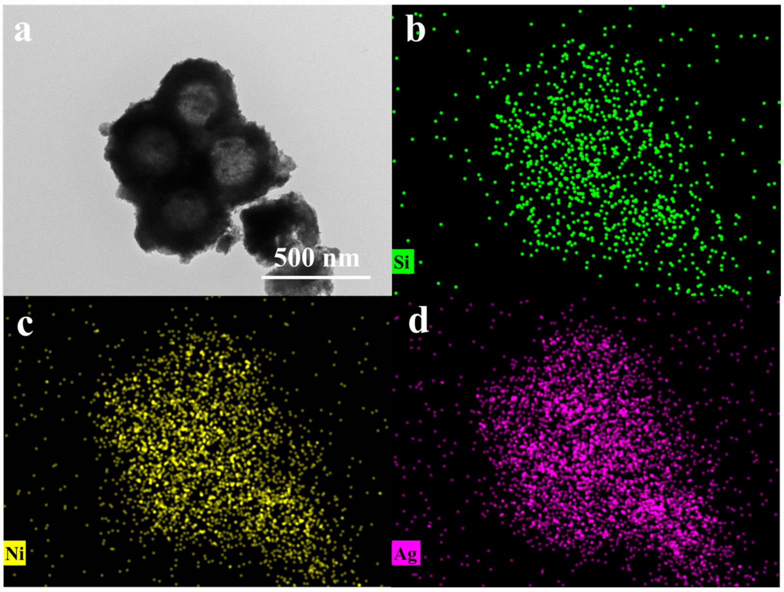

Fig.S5 (a) TEM image of Ag/NiSi-Ni and their EDS elemental mappings of Si(b), Ni(c) and Ag(d) elements.

2024-08-23 21:24:22 Admin

Full Analysis-AtmHe34mm

Sum	Compton	Ni	Ag	Si
	80.4 KCps	603.0 KCps	328.6 KCps	8.5 KCps
56.32	61.4 %	42.0 %	40.7 %	17.4 %

Fig.S6 XRF test results of Ag/NiSi-Ni.

Table S1. Comparison of the performance of Coercivity (Oe)

Sample	Coercivity (Oe)	Ref.
Annealing steel	0.45	1
AISI 304 Steel	63	2
Co/Pt(111)	0.1	3
IPM Machine	168	4
Ag/NiSi-Ni	383.4	This work

Table S2. Comparison of the performance of Resistivity (Ω m).

Sample	Resistivity (Ω m)	Ref.
H_2SO_4@MIL-101-SO_3H	5.5×10^{-3}	5
CS/H_2SO_4@MIL-101-8	1.1×10^{-1}	6
PIL@MIL	2.7×10^{-1}	7
ImPEEK/ImMIL-101(Cr)	2.1×10^{-1}	8
BUT-8(Cr)A	7.9×10^{-1}	9
DNA@ZIF-8	5.9×10^{-2}	10
Ag/NiSi-Ni	1.2×10^{-3}	This work

References

[1] Petryshynets, I.; Kovac, F.; Stoyka, V.; Boruta, J.; Influence of microstructure evolution on the coercive forces in low silicon non-oriented steels. Acta Phys. Pol. A 2010, 118, 1013-1014.

[2] Gopkalo, O.; Bezlyudko, G.; Nekhotiashchiy, V.; Gopkalo, O.; Kurash, Y.; Damage Evaluation For AISI 304 Steel Under Cyclic Loading Based On Co-ercive Force Measurements. 2020, 139, 105752.

[3] Chang, C.H.T.; Kuo, W.H.; Chang, Y.C.; Tsay, J.S.; Yau, S.L.; Tuning coercive force by adjusting electric potential in solution processed Co/Pt(111) and the mechanism involved. Sci. Rep. 2017, 7, 43700.

[4] Kato, T.; Limsuwan, N.; Yu, C;Y.; Akatsu, K.; Lorenz, R;D.; Rare earth reduction using a novel variable magnetomotive force flux-Intensified IPM machine. IEEE Trans. Ind. Appl. 2014, 50, 1748-1756.

[5]Tsai, M.K.; Chiu, R.; He, E.R.; Chen, J.Y.; Chu, F.; Tsai, J.; Wang, Y.P.; Jian, S.Y.; Chen, S.M. Innovative EMI Shielding Solutions on Advanced SiP Module for 5G Application. ELECTRONICS PACKAGING TECHNOLOGY CONFERENCE 2019,

601-607.
[6] Zeng, S.; Huang, Z.X.; Jiang, H.; Li, Y. From Waste to Wealth: A Lightweight and Flexible Leather Solid Waste/Polyvinyl Alcohol/Silver Paper for Highly Efficient Electromagnetic Interference Shielding. ACS Appl. Mater. Interfaces 2020, 12, 52038-52049.
[7] Li, Z; Wang, W.Y.; Chen, Y.J.; Xiong, C.Y.; He, G.W.; Cao, Y.; Wu, H.; Guiver, M.D.; Jiang, Z.Y.; Constructing efficient ion nanochannels in alkaline anion exchange membranes by in-situ assembly of poly (ionic liquid) in metal-organic frameworks. J. Mater. Chem. A 2016, 4, 2340-2348.
[8] He, X.Y.; Gang, M.Y.; Li, Z.; He, G.W.; Yin, Y.H.; Cao, L.; Zhang, B.; Wu, H.; Jiang, Z.Y.; Highly conductive and robust composite anion exchange membranes by incur porating quaternized MIL-101(Cr). Sci. Bull. 2017, 62, 266-276.
[9] Yang, F.; Xu, G.; Dou, Y;B.; Wang, B.; Zhang, H.; Wu, H.; Zhou, W.; Li, J.R.; Chen, B.L.;A flexible metal-organic framework with a high density of sulfonic acid sites for proton conduction. Nat. Energy 2017, 2, 877-883.
[10] Guo, Y.; Jiang, ZQ.; Ying, W.; Chen, LP.; Liu, YZ.; Wang, XB.; Jiang, ZJ.; Chen, BL.; Peng, XS.; DNA-Threaded ZIF-8 Membrane with High Proton Conductivity and Low Methanol Permeability. Adv. Mater. 2018, 30, 1705155.
